# Adverse events associated with ustekinumab in Crohn's disease treatment: an analysis based on the FAERS database

**DOI:** 10.3389/fmed.2025.1657247

**Published:** 2025-11-06

**Authors:** Chiwei Guo, Shujuan Zhao

**Affiliations:** 1 Department of Pharmacy, Henan Provincial People's Hospital, People's Hospital of Zhengzhou University, School of Clinical Medicine, Henan University, Zhengzhou, Henan, China; 2 Department of Endocrinology, The First People's Hospital of Nankang District, Ganzhou, Jiangxi, China

**Keywords:** Crohn's disease, adverse events, FAERS, ustekinumab, treatment

## Abstract

**Background:**

Ustekinumab, approved in 2016 for the treatment of Crohn's disease (CD), its safety profile remains insufficiently characterized in real-world settings. This study aims to enhance clinical safety by identifying adverse events (AEs) associated with ustekinumab through data mining of the U.S. Food and Drug Administration Adverse Event Reporting System (FAERS) database.

**Methods:**

AEs data for ustekinumab in CD treatment were extracted from the FAERS database. Duplicate and incomplete reports were excluded during preprocessing. Signal detection was performed using the reported odds ratio (ROR), proportional reporting ratio (PRR), Bayesian confidence propagation neural network (BCPNN), and empirical Bayesian geometric mean (EBGM), with established thresholds applied for signal identification. AEs were classified based on the system organclass (SOC).

**Results:**

A total of 17,187 AEs associated with ustekinumab in CD were identified, generating 44,232 signals across 24 SOCs and 258 preferred terms (PTs). The most frequent reports were categorized under “injury, poisoning, and procedural complications,” while “infections and infestations” emerged as one of the most frequently reported SOCs. Statistically significant PTs included abscess (ROR = 25.36, 95% CI: 22.51–28.58), clostridium difficile infection (ROR = 14.37, 95% CI: 12.72–16.24), and lower respiratory tract infection (ROR = 13.54, 95% CI: 12.37–14.83). Notable signals were also identified for hepatobiliary disorders (2.24% mortality) and cardiac disorders (7.50% mortality, the highest among all SOCs). Rare events, such as congenital pulmonary airway malformation, were observed; however, these findings were limited to a small number of cases and warrant cautious interpretation.

**Conclusion:**

In the treatment of CD with ustekinumab, it is critical to monitor not only common AEs like infections and tumors, but also less frequent yet severe AEs, including cardiac disorders, hepatobiliary disorders, and possible congenital anomalies. The latter, however, requires further validation through additional clinical evidence.

## 1 Introduction

Crohn's disease (CD) is a chronic inflammatory bowel disease that affects the gastrointestinal tract, with a globally rising incidence. In developed regions such as North America and Europe, disease prevalence exceeds 0.3%, and it is steadily increasing in newly industrialized countries, contributing to a significant global disease burden. Globally, Crohn's disease profoundly compromises the quality of life for those affected and places a substantial annual economic burden on societies (1–3). The pathogenesis of CD is believed to involve immune and microbial dysregulation, among other mechanisms, though the precise details remain incompletely understood. Insights into these mechanisms have driven the development of targeted therapeutic strategies, including hormonal therapies, immunotherapies, and biologic treatments (4, 5).

Ustekinumab, a monoclonal antibody targeting the shared p40 subunit of interleukins 12 and 23 (IL-12 and IL-23), acts by inhibiting inflammatory pathways central to CD pathogenesis (6). In 2016, the United States Food and Drug Administration (FDA) approved ustekinumab for the treatment of CD, following clinical trials that demonstrated its superiority over placebo in achieving and maintaining clinical remission (7). This approval marked a significant advancement in therapeutic options for CD.

Despite its demonstrated efficacy, the safety profile of ustekinumab in the treatment of CD remains insufficiently characterized. The robust efficacy of ustekinumab in Crohn's disease is supported by clinical guidelines from several countries, including the UK and South Korea, and is further corroborated by positive clinical studies conducted globally (8–11). Current research primarily focuses on its efficacy and safety, positioning ustekinumab as an alternative treatment option for Crohn's disease following the failure of traditional immunosuppressive therapy or tumor necrosis factor-alpha (TNF-α) antagonist treatment (12). As the use of ustekinumab for Crohn's disease is relatively recent, its long-term safety profile is still evolving. Emerging evidence from clinical trials and post-marketing surveillance has begun to characterize the associated adverse events (8, 9). However, while clinical trials provide critical safety data, they are limited by sample size, trial duration, and population diversity. Pharmacovigilance studies, which analyze real-world safety signals, are essential to identify rare adverse events and reactions in patient populations not typically represented in clinical trials. Such analyses are especially valuable for biologics like ustekinumab.

The U.S. Food and Drug Administration Adverse Event Reporting System (FAERS) is a publicly accessible database that aggregates reports of adverse drug events submitted by healthcare professionals, patients, and pharmaceutical manufacturers. This resource allows researchers to identify potential adverse event (AE) signals and assess associated risks (13). Disproportionality analysis methods, commonly used in pharmacovigilance research, are essential for detecting signals that merit further investigation.

In this study, we analyzed the FAERS database to identify AE signals associated with ustekinumab use in CD. Four established statistical methods for signal detection were employed, including reported odds ratio (ROR), proportional reporting ratio (PRR), Bayesian confidence propagation neural network (BCPNN), and empirical Bayesian geometric mean (EBGM) (14). The objective was to characterize adverse event signals of ustekinumab in Crohn's disease, providing valuable insights to guide clinical monitoring and inform potential updates to drug labeling.

## 2 Methods

### 2.1 Data source and analysis

#### 2.1.1 Source of data

This study utilized AE data from the FAERS, an internationally recognized and globally accessible pharmacovigilance database. Established in 2004, the FAERS database is characterized by its large scale, high standardization, and quarterly updates. It compiles AE reports submitted by healthcare professionals, pharmaceutical companies, patients, and other sources.

Given that ustekinumab was approved by the FDA for the treatment of CD on September 23, 2016, this study retrieved relevant AE reports from the FAERS database spanning from Q4 2016 to Q4 2023. Data extraction and preprocessing were conducted using R language (version 4.4.0). Signal detection analyses were performed with custom R scripts and proprietary algorithms developed by the research team, ensuring methodological rigor and reproducibility.

#### 2.1.2 Standardization of drug names and adverse drug reactions

In this study, ustekinumab was classified as the suspected drug type, and its name was standardized using the Medex_UIMA_1.3.8 tool to ensure consistency. To focus exclusively on CD, the dataset was restricted to reports where CD was documented as the indication for ustekinumab in the INDICATIONS_PT field of the FAERS database. Reports related to other approved indications of ustekinumab were excluded to maintain specificity.

Data preprocessing involved removing duplicate reports to achieve a standardized dataset. Duplicate entries were identified using the FDA's unique case identifiers, with further verification through patient demographics and event dates when necessary. The latest version of the Medical Dictionary for Regulatory Activities (MedDRA 26.1) (15) was employed to map AEs to their corresponding preferred terms (PTs) and system organ classes (SOCs).

Demographic variables extracted from the database included age, gender, reporting country, reporter type, occurrence time, and clinical outcomes, providing a comprehensive view of the reported events. Descriptive statistical analyses were conducted on these variables to identify potential patterns in adverse event reporting.

### 2.2 Data signal analysis

This study employed commonly used asymmetrical measurements in pharmacovigilance research to identify potential signals between AEs and the treatment of CD with ustekinumab. These data mining techniques analyze the correlation between drugs and AEs by comparing observed frequencies in populations exposed and unexposed to the drug, utilizing a four-grid table (Supplementary Table S1).

Four established signal detection methods were applied: ROR (16), PRR (17), BCPNN (18), and EBGM (19). The BCPNN algorithm primarily evaluates the association strength between drugs and adverse reactions using the information component (IC) and its 95% confidence interval (CI), based on classical four-fold tables and Bayesian discrimination principles. This method is particularly effective for the early detection of adverse event signals.

The parameters for AE signal detection were defined as follows (Supplementary Table S2): (1) for ROR, when a ≥ 3, ROR ≥ 3 and the lower bound of the 95% CI > 1; (2) for PRR, when a ≥ 3, PRR ≥ 2 and the lower bound of the 95% CI > 1; (3) for BCPNN, when the lower limit of the IC > 0; and (4) for EBGM, when the lower limit of the 95% CI for the EBGM > 2 (20, 21). In these disproportionality methods, “a” represents the number of occurrences of the target AE for the target drug. In this study, we combined the ROR, PRR, BCPNN, and MGPS algorithms. By combining the ROR, PRR, BCPNN, and EBGM methods, this study aimed to leverage the strengths of each algorithm, broaden the detection scope, and cross-validate results from multiple perspectives.

**Figure 1:**
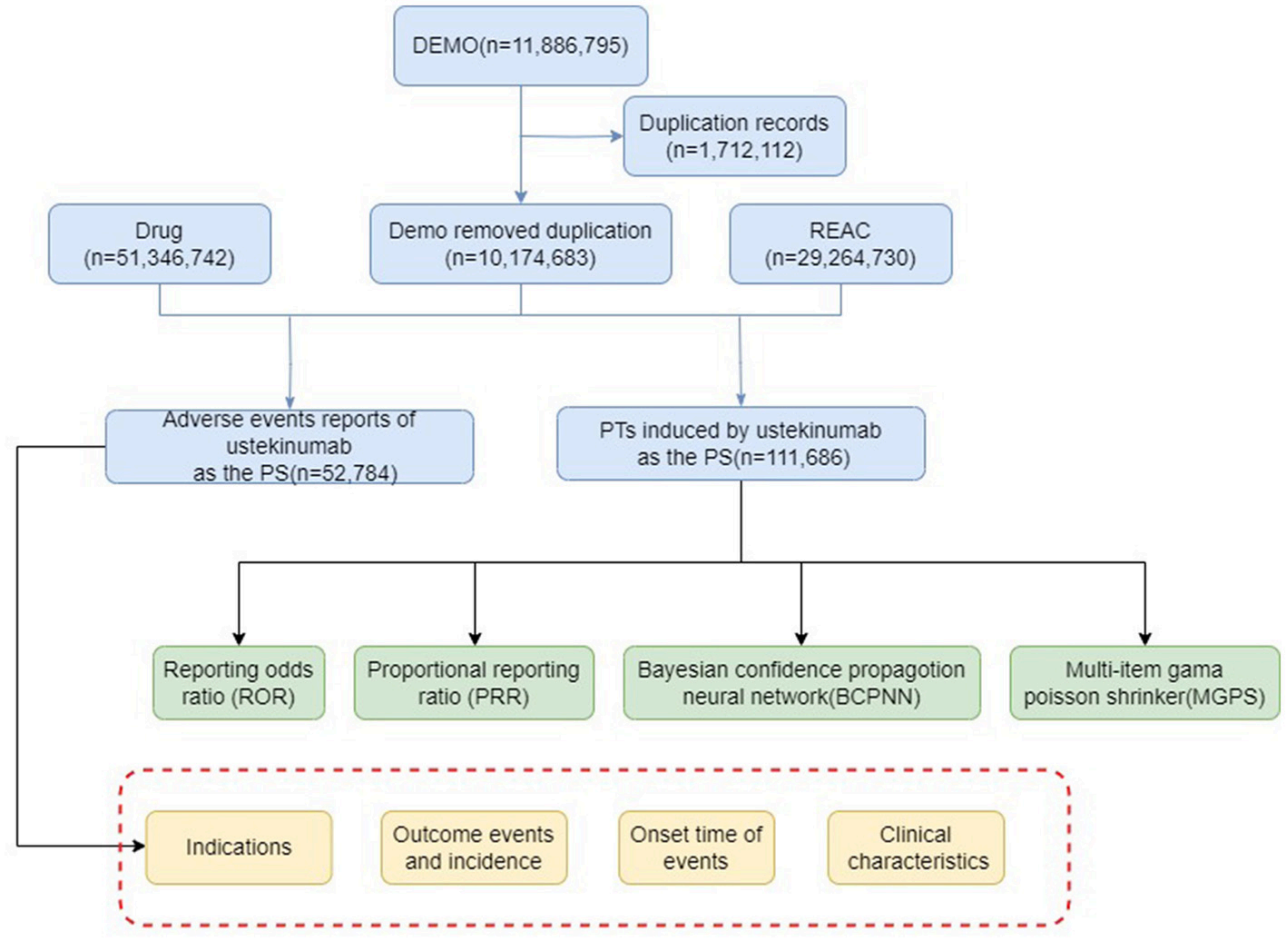
The flow diagram of selecting ustekinumab-related AEs from FAERS database.

AE results were considered effective if they met the positive signal criteria for all four algorithms. All data related to AEs associated with ustekinumab in the treatment of CD were processed and statistically analyzed using software such as RStudio (version 4.4.0). The overall workflow of the study is illustrated in Figure 1.

## 3 Results

### 3.1 Basic information of patients with adverse events related to ustekinumab in Crohn's disease treatment

A total of 10,573,648 AE reports were extracted from the FAERS database, of which 47,494 were related to ustekinumab. After restricting the indication to CD and the timeframe to Q4 2016–Q4 2023, 17,187 AE reports were identified, encompassing 44,232 AEs across 24 SOC categories. Of these, 258 PTs exhibited positive signals according to all four signal detection algorithms.

The demographic and reporting characteristics of the AEs are summarized in Table 1. The highest number of reports was recorded in 2020 (28.22%). Female patients accounted for the majority (58.14%), and the 30 to 39 age group was the most represented (12.31%). Most reports originated from the United States (54.87%), with patients (41.68%) and pharmacists (34.32%) being the primary reporters. Subcutaneous injection was the predominant route of administration (80.35%).

**Table 1:** Basic information of ulinumab with Crohn's disease in the FAERS database.

**Characteristics**	**Total no. (%)**	**Characteristics**	**Total no. (%)**
Number of events	17,187 (100)	Route	
Gender		Subcutaneous	13,810 (80.35)
Female	9,993 (58.14)	Intravenous	2,352 (13.68)
Male	6,008 (34.96)	Outcomes	
Not specified	1,186 (6.90)	Other serious	8,040 (63.82)
Age (years)		Hospitalization	3.965 (31.48)
Median (IQR)	45 (31–59)	Death	232 (1.84)
< 20 years	629 (3.66)	Life-threatening	206 (1.64)
20–29 years	1,663 (9.68)	TTO (IQR)	80 (0–347)
30–39 years	2,023 (11.77)	< 7	1,194 (10.87)
40–49 years	1,998(11.63)	7–28	233 (2.12)
50–59 years	1,966 (11.44)	28–60	370 (3.37)
≥ 60 years	2,546 (14.81)	≥ 60	2,043 (18.59)
Not specified	6,362 (37.02)	Not specified	7,147 (65.05)
		Reporting year	
Reporter		2016	59 (0.34)
Consumer	7,163 (41.68)	2017	895 (5.21)
Pharmacist	5,898 (34.32)	2018	1,962 (11.42)
Physician	2,601 (15.13)	2019	2,319 (13.49)
Other health-professional	1,320 (7.68)	2020	4,851 (28.22)
Reported countries (top 5)		2021	2,298 (13.37)
United States	9,430 (54.87)	2022	2,406 (14.00)
Canada	3,841 (22.35)	2023	2,397 (13.95)
United Kingdom	13.99 (8,14)		
Other	372 (2.16)		
Australia	307 (1.79)		

FAERS, the U.S. Food and Drug Administration Adverse Event Reporting System; IQR, interquartile range.

**Figure 2:**
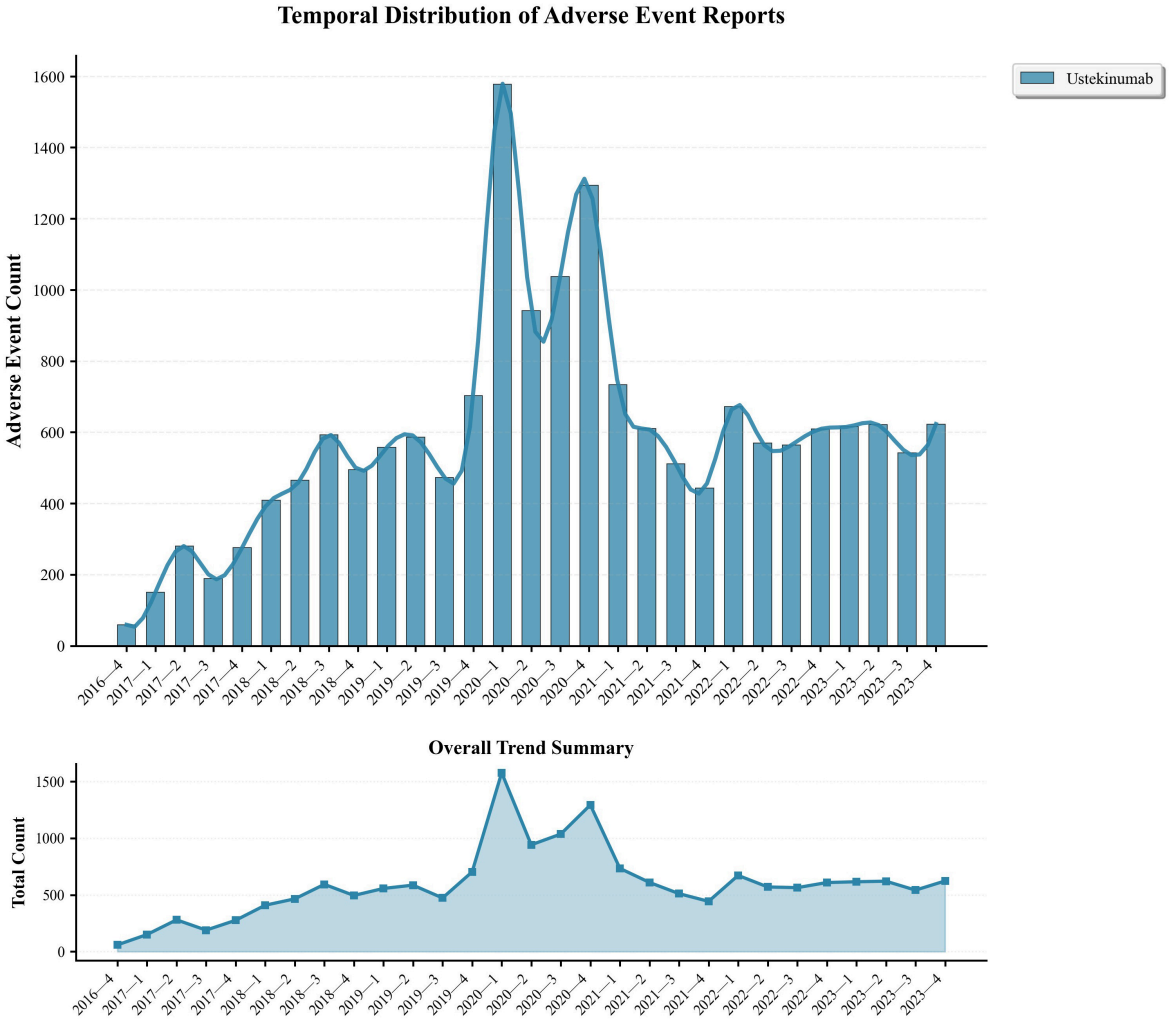
Temporal distribution and trend of adverse event reports.

Serious adverse outcomes included hospitalization (31.48%), death (1.84%), and life-threatening events (1.64%), while a significant proportion of reports had unknown serious outcomes (63.82%). AEs showed an increasing trend from 2016, peaking in 2020 (Figure 2). Following this demographic analysis, specific AE signals associated with ustekinumab in CD treatment were examined.

### 3.2 Signal detection related to ustekinumab in Crohn's disease treatment

#### 3.2.1 Signal detection based on SOC

**Figure 3:**
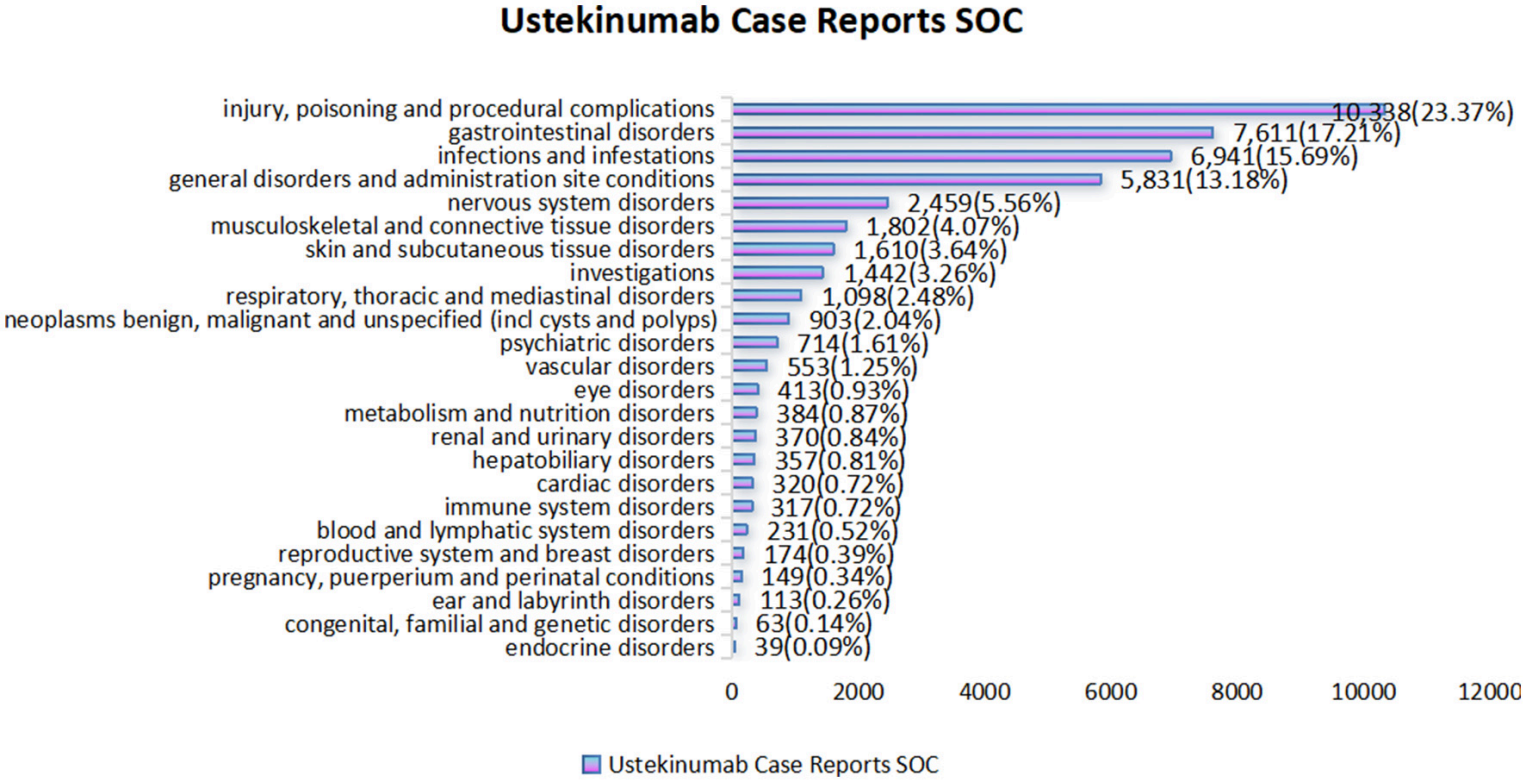
Statistical chart of the number and percentage of 24 SOC cases reported in cases related to the treatment of Crohn's disease with ustekinumab.

The analysis identified 24 SOCs associated with AEs in CD treatment using ustekinumab (Figure 3). The top three reported SOCs were: injury, poisoning, and procedural complications (*n* = 10,338, 23.37%), gastrointestinal disorders (*n* = 7,611, 17.21%), and infections and infestations (*n* = 6,941, 15.69%). Infections and infestations were identified as positive signals by all four detection methods, aligning with ustekinumab's known safety profile as outlined in its prescribing information. Other SOCs, such as vascular disorders (*n* = 553, 1.25%), eye disorders (*n* = 413, 0.93%), cardiac disorders (*n* = 320, 0.72%), and metabolism and nutrition disorders (*n* = 384, 0.87%), were not listed in the drug's label but warrant attention for potential preventive measures. To gain more detailed clinical insights, we further analyzed the adverse events at the PT level.

#### 3.2.2 Signal detection based on PT levels

At the PT level, 258 signals were identified using all four algorithms. The top 30 signals, ranked by report frequency and ROR, are presented in Tables 2 and 3. The analysis prioritized biologically plausible signals with clinical relevance. Significant signals included: abscess (ROR=25.36, 95% CI: 22.51–28.58; IC025 = 4.43; EBGM05 = 22.01), clostridium difficile infection (ROR = 14.37, 95% CI: 12.72–16.24; IC025 = 3.63; EBGM05 = 12.65), and lower respiratory tract infection (ROR = 13.54, 95% CI: 12.37–14.83; IC025 = 3.59; EBGM05 = 12.2). Other important signals included intestinal obstruction (ROR = 10.01, 95% CI: 8.87–11.29; IC025 = 3.12; EBGM05 = 8.88), fistula (ROR = 25.69, 95% CI: 22.29–29.6; IC025 = 4.42; EBGM05 = 21.9), and anal abscess (ROR = 40.66, 95% CI: 34.88–47.4; IC025 = 5.04; EBGM05 = 33.62).

**Table 2:** Biologically significant signals of ustekinumab in Crohn's disease ranked by number of reports using disproportionality analysis among the top 30 PTs.

**PTs**	**AE Reports**	**ROR (95% Cl)**	**PRR (95% Cl)**	**BCPNN IC (IC025)**	**EBGM (EBGM05)**
Abdominal pain	694	4.42 (4.1, 4.77)	4.37 (4.04, 4.73)	2.12 (2.01)	4.35 (4.08)
Lower respiratory tract infection	479	13.54 (12.37, 14.83)	13.41 (12.16, 14.79)	3.72 (3.59)	13.16 (12.2)
Infusion related reaction	453	8.99 (8.19, 9.87)	8.91 (8.08, 9.83)	3.14 (3)	8.8 (8.14)
Abscess	282	25.36 (22.51, 28.58)	25.21 (22.41, 28.36)	4.6 (4.43)	24.32 (22.01)
Intestinal obstruction	269	10.01 (8.87, 11.29)	9.95 (8.85, 11.19)	3.3 (3.12)	9.82 (8.88)
Clostridium difficile infection	264	14.37 (12.72, 16.24)	14.29 (12.7, 16.07)	3.81 (3.63)	14.01 (12.65)
Fistula	199	25.69 (22.29, 29.6)	25.58 (22.3, 29.34)	4.62 (4.42)	24.66 (21.9)
Haematochezia	193	4.41 (3.82, 5.08)	4.39 (3.83, 5.04)	2.13 (1.92)	4.37 (3.88)
Anal abscess	174	40.66 (34.88, 47.4)	40.5 (34.62, 47.38)	5.26 (5.04)	38.22 (33.62)
Frequent bowel movements	160	7.6 (6.5, 8.88)	7.57 (6.47, 8.86)	2.91 (2.68)	7.5 (6.58)
Drug level decreased	147	16.71 (14.18, 19.68)	16.65 (14.23, 19.48)	4.02 (3.79)	16.27 (14.18)
Cellulitis	132	3.59 (3.03, 4.27)	3.59 (3.01, 4.28)	1.84 (1.59)	3.57 (3.1)
Kidney infection	124	7.97 (6.68, 9.52)	7.95 (6.66, 9.48)	2.98 (2.72)	7.87 (6.78)
Nephrolithiasis	114	3.36 (2.8, 4.04)	3.36 (2.82, 4.01)	1.74 (1.48)	3.35 (2.87)
Intestinal stenosis	102	35.43 (29.03, 43.25)	35.35 (29.06, 43)	5.07 (4.78)	33.61 (28.44)
Abdominal abscess	84	28.58 (22.97, 35.57)	28.53 (23, 35.39)	4.78 (4.46)	27.39 (22.81)
Gastrointestinal infection	79	11.95 (9.57, 14.93)	11.93 (9.62, 14.8)	3.55 (3.23)	11.74 (9.74)
Skin cancer	78	3.77 (3.02, 4.71)	3.77 (3.04, 4.68)	1.91 (1.59)	3.75 (3.11)
Postoperative wound infection	76	13.73 (10.94, 17.23)	13.71 (10.84, 17.35)	3.75 (3.42)	13.45 (11.12)

PT, preferred term; AE, adverse event; FAERS, the U.S. Food and Drug Administration Adverse Event Reporting System; ROR, reporting odds ratio; CI, confidence interval; PRR, proportional reporting ratio; BCPNN, Bayesian confidence propagation neural network; IC, information component; EBGM, empirical Bayesian geometric mean.

**Table 3:** Biologically significant signals of ustekinumab in Crohn's disease ranked by ROR using disproportionality analysis among the top 30 PTs.

**PTs**	**AE reports**	**ROR (95% Cl)**	**PRR (95% Cl)**	**BCPNN IC (IC025)**	**EBGM (EBGM05)**
Transitional cell carcinoma recurrent	3	99.1 (29.45, 333.51)	99.09 (29.4, 334.03)	6.43 (4.9)	86.3 (31.26)
Spirochaetal infection	3	90.09 (26.96, 301.01)	90.08 (26.72, 303.66)	6.31 (4.79)	79.39 (28.93)
Fecal calprotectin	4	82.58 (29.2, 233.53)	82.58 (29.22, 233.36)	6.2 (4.85)	73.51 (30.81)
Infected fistula	36	80.14 (56.7, 113.27)	80.08 (56.27, 113.96)	6.16 (5.67)	71.53 (53.55)
External ear cellulitis	6	66.07 (28.54, 152.93)	66.06 (28.44, 153.45)	5.91 (4.79)	60.15 (29.8)
Periumbilical abscess	3	61.94 (18.97, 202.27)	61.93 (19.11, 200.74)	5.83 (4.34)	56.71 (21.07)
Anal fistula infection	8	56.84 (27.6, 117.03)	56.83 (27.52, 117.36)	5.71 (4.73)	52.41 (28.64)
Small intestine adenocarcinoma	7	55.06 (25.47, 119.05)	55.05 (25.63, 118.23)	5.67 (4.63)	50.89 (26.7)
Rectal abscess	67	54.32 (42.34, 69.7)	54.24 (42.04, 69.98)	5.65 (5.29)	50.2 (40.75)
Abscess intestinal	70	49.38 (38.72, 62.96)	49.3 (38.97, 62.37)	5.52 (5.17)	45.95 (37.49)
Incision site abscess	7	48.68 (22.59, 104.9)	48.68 (22.67, 104.55)	5.5 (4.47)	45.41 (23.89)
Bartholin's abscess	3	47.19 (14.63, 152.25)	47.19 (14.56, 152.96)	5.46 (3.99)	44.11 (16.55)
Jejunal stenosis	3	46.09 (14.3, 148.58)	46.09 (14.22, 149.4)	5.43 (3.96)	43.15 (16.2)
Postoperative abscess	21	44.34 (28.5, 68.98)	44.32 (28.24, 69.56)	5.38 (4.76)	41.6 (28.74)
Anastomotic stenosis	10	44.05 (23.22, 83.56)	44.04 (23.06, 84.09)	5.37 (4.49)	41.35 (24.2)
Anal abscess	174	40.66 (34.88, 47.4)	40.5 (34.62, 47.38)	5.26 (5.04)	38.22 (33.62)
Vaginal fistula	10	37.54 (19.85, 71)	37.54 (19.66, 71.68)	5.15 (4.28)	35.57 (20.87)
Omphalitis	9	36.71 (18.76, 71.83)	36.7 (18.85, 71.46)	5.12 (4.2)	34.82 (19.86)
Perineal abscess	14	36 (21.02, 61.65)	35.99 (21.2, 61.1)	5.1 (4.34)	34.18 (21.79)
Intestinal stenosis	102	35.43 (29.03, 43.25)	35.35 (29.06, 43)	5.07 (4.78)	33.61 (28.44)
Choroid melanoma	3	35.39 (11.08, 113.08)	35.39 (11.13, 112.49)	5.07 (3.61)	33.64 (12.73)
Pyoderma	14	35.31 (20.62, 60.46)	35.3 (20.79, 59.92)	5.07 (4.32)	33.56 (21.4)
Abscess rupture	8	34.32 (16.86, 69.87)	34.32 (16.95, 69.5)	5.03 (4.06)	32.67 (18.03)
Abdominal wall abscess	13	32.66 (18.71, 57.01)	32.65 (18.86, 56.52)	4.96 (4.19)	31.16 (19.55)
Vaginal abscess	6	31.21 (13.76, 70.79)	31.21 (13.7, 71.09)	4.9 (3.8)	29.85 (15.04)
Arthritis enteropathic	7	30.03 (14.08, 64.06)	30.03 (13.98, 64.5)	4.85 (3.82)	28.77 (15.26)
Abdominal abscess	84	28.58 (22.97, 35.57)	28.53 (23, 35.39)	4.78 (4.46)	27.39 (22.81)
Stoma site abscess	6	26.43 (11.69, 59.77)	26.42 (11.6, 60.18)	4.67 (3.58)	25.45 (12.86)

PT, preferred term; AE, adverse event; FAERS, the U.S. Food and Drug Administration Adverse Event Reporting System; ROR, reporting odds ratio; CI, confidence interval; PRR, proportional reporting ratio; BCPNN, Bayesian confidence propagation neural network; IC, information component; EBGM, empirical Bayesian geometric mean.

Some signals with exceptionally high ROR values, such as congenital pulmonary airway malformation (ROR = 116.59, 95% CI: 34.17–397.85; IC025 = 5.09; EBGM05 = 35.54), were based on a small number of reports (*n* = 3), and should therefore be interpreted with caution. Other signals related to medication handling, such as product storage error, accidental exposure to product, and off-label use, may represent reporting artifacts rather than true AEs. The full list of identified PT signals is provided in Supplementary Tables S3 and S4. To better understand severe outcomes, fatal AE patterns were specifically analyzed.

#### 3.2.3 Signal analysis of fatal adverse events

Fatal AEs were identified across 17 SOCs. The leading causes of death were infectious diseases (136 cases, 1.96% mortality), systemic diseases (121 cases, 2.08%), and various injuries (60 cases, 0.58%). The highest mortality rates were observed in SOCs of cardiac disorders (7.50%), neoplasms (5.76%), and hepatobiliary system disorders (2.24%).

These mortality patterns align with the disproportionality analysis, where infections exhibited strong positive signals (e.g., abscess, ROR = 25.36), while cardiac and hepatobiliary disorders, despite fewer reports, were associated with disproportionately high fatality rates. Among these, only neoplasms were explicitly mentioned in the drug's prescribing information (Table 4).

**Table 4:** Total number and percentage of death.

**SOC**	**Death case reports**	**Total case reports**	**Death proportion**
Injury, poisoning and procedural complications	60	10,338	0.58%
Investigations	10	1,442	0.69%
Gastrointestinal disorders	59	7,611	0.78%
Musculoskeletal and connective tissue disorders	16	1,802	0.89%
Nervous system disorders	24	2,459	0.98%
Psychiatric disorders	7	714	0.98%
Immune system disorders	4	317	1.26%
Respiratory, thoracic and mediastinal disorders	17	1,098	1.55%
Metabolism and nutrition disorders	6	384	1.56%
Vascular disorders	9	553	1.63%
Renal and urinary disorders	7	370	1.89%
Infections and infestations	136	6,941	1.96%
General disorders and administration site conditions	121	5,831	2.08%
Blood and lymphatic system disorders	5	231	2.17%
Hepatobiliary disorders	8	357	2.24%
Neoplasms benign, malignant and unspecified (incl cysts and polyps)	52	903	5.76%
Cardiac disorders	24	320	7.50%

SOC, system organ class.

## 4 Discussion

This study analyzed AEs related to ustekinumab in the treatment of CD using data from the FAERS database, aiming to provide new clinical insights. As an antagonistic antibody targeting IL-12 and IL-23, ustekinumab modulates key cytokines implicated in cancer, infections, and inflammatory diseases, making careful monitoring of associated AEs critical (7, 22, 23). The consistency of our findings with the drug's established mechanism of action—especially the strong signals for infections detected across all four statistical methods—supports the reliability of our methodology and validates the known safety profile of ustekinumab.

### 4.1 Demographic characteristics of AEs related to ustekinumab in Crohn's disease treatment

Our data revealed that 58.14% of AE reports involved female patients, compared to 34.96% involving males. This observed gender distribution may reflect the geographical composition of the FAERS database, as most reports (54.87%) originated from the United States. Epidemiological studies indicate that CD is more prevalent in women in the United States, whereas it is more common in men in Japan (24). Without appropriate population-based denominators, it is difficult to determine whether this gender pattern represents a true difference in drug-related AE risks, or a reporting bias associated with regional population characteristics.

The limited representation of pediatric patients (3.66% under 20 years) highlights a key surveillance gap. This finding reflects an inherent limitation of the FAERS database, which is less sensitive in capturing AEs for rare subgroups such as children. Although retrospective studies suggest that ustekinumab is safe for pediatric patients (25, 26), our results underscore the need for dedicated pharmacovigilance studies targeting this understudied population. Similarly, older adults (14.81% above 60 years) were underrepresented in the dataset. While some reports show that ustekinumab is effective and safe for older patients with CD (27), the efficacy is comparable to other age groups, but the increased burden of comorbidities in the elderly requires a comprehensive management strategy, including thorough baseline assessment, vigilant monitoring of complications, and appropriate dose adjustments (28, 29). Larger, age-specific studies are required to confirm these findings.

The reliability of the FAERS data is strengthened by the fact that nearly half (49.45%) of the reports were submitted by healthcare professionals, who are generally more familiar with AE reporting protocols. However, the predominance of reports from developed countries exposes disparities in pharmacovigilance infrastructure globally. Hospitalization rates for CD are rising rapidly in less developed countries, whereas rates in North America and Western Europe have stabilized (30). This trend highlights the urgent need to establish robust adverse event monitoring systems in emerging regions to ensure global patient safety.

### 4.2 Known systemic adverse reactions and their new signals

Our analysis confirms the established safety profile of ustekinumab while identifying several new signals that warrant clinical attention. Infections were the most frequently reported AEs, mainly affecting the respiratory tract, with nasopharyngitis and upper respiratory tract infections being the most common. The malignancy profile was dominated by non-melanoma skin cancers (31), while other malignancies were reported at lower frequencies. Gastrointestinal AEs primarily manifested as vomiting, abdominal pain, and diarrhea (32–34). These findings are consistent with existing guidelines and prescribing information for ustekinumab.

Reported cases of CD exacerbation may reflect therapeutic failure rather than true AEs, consistent with the known phenomenon of efficacy attenuation in biological therapies. Previous studies have shown that dose escalation can restore clinical response in over 50% of patients who fail standard maintenance dosing (35).

Beyond confirming these known risks, our signal detection analysis revealed several potential new safety concerns. It is crucial to note that FAERS data suggest associations rather than causality, and these findings require further validation. New infection signals, including lower respiratory tract infections, spirochetal infections, mycoplasma infections, and oral infections, are biologically plausible given ustekinumab's immunomodulatory effects, but must be confirmed in clinical studies. Similarly, gastrointestinal signals such as ulceration, stenosis, and obstruction may reflect disease progression rather than direct drug effects.

The identification of these new adverse reaction signals carries significant clinical implications. Firstly, it alerts clinicians to consider a potential association with ustekinumab when patients present with corresponding symptoms during treatment, thereby facilitating accurate diagnosis. This is particularly relevant for high-risk individuals, in whom a more thorough risk-benefit assessment is warranted prior to prescription. Secondly, patient management strategies should be enhanced through education on recognizing and reporting these potential events, a process that can be supported by pharmacists during medication dispensing. Finally, these findings provide real-world evidence to pharmacovigilance agencies, suggesting that such reactions be prioritized for active monitoring and considered for inclusion in future updates to the drug's prescribing information. Collectively, these measures will contribute to earlier risk identification, more timely intervention, and improved patient safety.

Signals for lymphoma and neuroendocrine tumors align with concerns about immunosuppression, and partially corroborate prior reports of lymphoma in CD patients treated with ustekinumab (36). This may be related to the mechanism of IL-23 pathway, the role of IL-23 in cancer is complex and context-dependent. It can exhibit both anti-tumor effects by potentiating cytotoxic T cells and pro-tumor activity via autocrine TGF-β signaling (37, 38). This duality is reflected in genetic and preclinical data: IL-23R polymorphisms may confer varying cancer risks (37), and IL-23 deficiency in mice increases susceptibility to certain tumors, likely by impairing immune surveillance and cellular homeostasis (39, 40). Our clinical findings are consistent with this complexity, showing significant adverse event signals in cancer associated with inhibitors of the IL-23 pathway. This highlights the necessity of individualized cancer risk assessment prior to biologic therapy. These findings suggest a need for heightened vigilance, but causality should be confirmed through controlled epidemiological studies before altering clinical practice.

Notably, procedure-related complications represented the largest category of adverse events (23.37%), suggesting that issues related to drug administration or reporting practices may contribute more significantly to this category than true pharmacological toxicity. This highlights the importance of standardized administration protocols and thorough patient education by healthcare providers to minimize preventable adverse outcomes.

### 4.3 Emerging systemic adverse reaction signals

While infections, gastrointestinal effects, and neoplasms are well-documented in ustekinumab's prescribing information, our signal detection also identified vascular, ocular, cardiac, metabolic, reproductive, and hepatobiliary signals. These findings suggest that AEs affecting other systems are emerging and warrant further investigation.

The signal for congenital pulmonary airway malformation deserves cautious interpretation. Despite its high ROR ranking, this signal is based on an extremely limited number of cases (*n* = 3), raising the possibility of a reporting artifact rather than true causality. Theoretically, a biological mechanism exists via placental transfer, as pharmacokinetic studies have shown significantly higher ustekinumab concentrations in umbilical cord blood compared to maternal serum (41). However, the current evidence on ustekinumab use during pregnancy is limited to isolated case reports, small observational studies, and spontaneous reports, with no well-designed prospective studies specifically examining congenital outcomes.

The conflicting evidence regarding pregnancy outcomes further underscores the need for caution. One case report documented miscarriage (42), while other studies reported favorable pregnancy outcomes (43). These inconsistencies underscore the limitations of the current evidence and the urgent need for prospective pregnancy cohort studies. Given the critical implications for maternal and fetal health, clinicians must carefully weigh these uncertainties in the risk-benefit assessment of ustekinumab (44, 45). Furthermore, therapeutic drug monitoring in both pregnant women and their newborns is essential until more conclusive evidence is available.

The signal for elevated fecal calprotectin presents an interesting case of biomarker fluctuations that may reflect disease activity monitoring rather than a true AE. Fecal calprotectin levels correlate with CD activity (46), and changes in this biomarker may indicate treatment response patterns rather than drug-related toxicity (47). This distinction between disease manifestations, treatment efficacy markers, and true AEs highlights the complexities of interpreting disproportionality analyses in chronic inflammatory conditions. For such diseases, longitudinal monitoring is crucial to distinguish between therapeutic effects and AEs accurately.

### 4.4 Death signal

This study included an analysis of death signals, with fatalities accounting for 1.84% of serious adverse events. Infections contributed the highest number of deaths, while the highest mortality rate was associated with cardiovascular system diseases (7.50%), including myocardial infarction and heart failure. This apparent paradox, relatively weak signal strength but high case-fatality, warrants particular clinical attention.

Although the current literature suggests a low mortality rate associated with ustekinumab, and case reports alone (e.g., intrauterine death) cannot establish causality, the considerable number of fatal outcomes observed in our substantial study cohort warrants careful consideration (42, 48).

A clinical study on ustekinumab for inflammatory bowel disease previously suggested the potential for cardiovascular AEs (33). Supporting this concern, a case report described a male Crohn's disease patient who developed heart failure after 10 months of ustekinumab treatment, with symptom improvement and restoration of cardiac function following drug discontinuation (49). Conversely, a meta-analysis found no increased risk of cardiovascular events with ustekinumab compared to placebo (50). Clinical studies have reported an association between ustekinumab use and a significant number of serious cardiovascular events, including acute coronary syndrome, ischemic stroke, and cardiovascular death. From the current research mechanism, cardiovascular AEs are related to the interleukin-17A (IL-17A) pathway, IL-17A may exert a stabilizing effect on atherosclerotic plaques by stimulating type I collagen production from smooth muscle cells within the fibrous cap and reducing the endothelial expression of vascular cell adhesion molecule-1 (VCAM-1), thereby limiting the recruitment of inflammatory cells. In contrast, ustekinumab, by inhibiting IL-23 (a key cytokine for TH17 cell homeostasis), may attenuate this protective pathway. This potential disruption of plaque stability could accelerate atherosclerosis, potentially leading to major adverse cardiovascular events and increased mortality (51–55). However, the precise pathophysiological mechanisms connecting IL-12/23 p40 inhibition to cardiovascular events are not fully defined. Further research is essential to clarify the specific contributions of the IL-12 and IL-23 pathways. This discrepancy between our pharmacovigilance findings and controlled trial data highlights the limitations of spontaneous reporting systems, which can be influenced by reporting biases, confounding factors, and missing data. These limitations underscore the need for further research to confirm and better understand these potential cardiovascular risks.

Infections represented the most common cause of death among these AEs. This fatal risk is mechanistically linked to the suppression of the IL-23/IL-17 pathway. Ustekinumab, by inhibiting IL-23, impairs the immune functions of IL-17A—a key cytokine in innate immunity that recruits neutrophils and synergizes with other pro-inflammatory factors (e.g., TNF-α, IL-1β, IL-22) to combat extracellular pathogens. Such immunosuppression may compromise host defense, leading to severe infections and fatal outcomes in certain patients (56). Therefore, a comprehensive infection management strategy for ustekinumab therapy is essential. Prior to initiation, this includes risk assessment and screening for latent tuberculosis, viral hepatitis, and ensuring vaccination status is updated. During treatment, vigilant monitoring and patient education on symptom recognition are crucial to facilitate early detection. This approach allows for the timely management of both mild and severe/opportunistic infections, which may necessitate temporary drug interruption or adjustment of the treatment regimen.

Despite the weak cardiovascular signal strength detected across the four statistical methods used in this study, the high case-fatality rate emphasizes the importance of vigilance to mitigate mortality risks. In addition, hepatobiliary disorders exhibited the third-highest mortality rate (2.24%), with signals for cholangitis and cholelithiasis. These signals were consistent across all four disproportionality methods employed in this study. However, it is important to acknowledge that FAERS data cannot establish true incidence or absolute risk due to the lack of a reliable denominator and the potential for reporting biases.

Nevertheless, the concerning mortality rate associated with hepatobiliary disorders highlights a critical gap in clinical trial data regarding this risk. Previous pre-marketing studies have not adequately addressed this issue (57), underscoring the value of pharmacovigilance in identifying serious risks that may be missed in controlled trials. These findings emphasize the need for heightened clinical awareness and further investigation to refine risk management strategies for ustekinumab treatment.

### 4.5 Limitation

This study has several limitations that should be acknowledged. First, the FAERS database is a self-reporting system, which is subject to missing, duplicate, inaccurate, incomplete reports, and reporting bias. These factors may introduce bias into the research findings. Second, the geographical distribution of reports is not comprehensive, leading to regional bias in the data. Third, some PT-level signals, such as congenital malformations, product problems, and accidental exposures, may represent reporting artifacts rather than true biological effects. These findings require cautious interpretation and further validation before causality can be established.

Fourth, the FAERS database does not reliably capture temporal relationships between drug exposure and the onset of AEs, and the severity of AEs reported by different reporters, limiting the ability to infer causality. Fifth, in the context of CD, distinguishing between disease manifestations, complications of the disease itself, and true drug-related AEs is particularly challenging. Finally, the database lacks phase-specific safety evaluations of the drug, as it only includes AE data reported since the drug's market launch.

To address these limitations, future studies should incorporate demographic stratification (e.g., by age, sex, and region), sensitivity analyses using different reporting sources, and more rigorous validation through registry-based or prospective cohort studies. Additionally, epidemiological approaches and temporal analyses are needed to provide more robust evidence regarding the safety profile of ustekinumab.

## 5 Conclusion

By utilizing the FAERS database, this study has identified both established and emerging AE signals associated with ustekinumab in the treatment of CD. While known AEs such as infections and lymphomas remain significant, new signals have emerged in other systems, including the cardiovascular, metabolic, reproductive, and genetic systems. Of particular concern are cardiac-related signals, despite their low signal strength, exhibiting a high mortality rate and requiring vigilant clinical monitoring. These findings emphasize the importance of close monitoring, not only for common infectious complications, but also for serious AEs affecting the heart and malignancies. Furthermore, this study underscores the need to recognize the risks associated with ustekinumab, particularly its potential impact on congenital and familial genetic diseases. Appropriate regulation of drug use and enhanced patient monitoring are essential to minimize the occurrence and severity of AEs.

Although randomized controlled trials (RCTs) have established the efficacy and safety of ustekinumab, their generalizability to broader, more heterogeneous real-world populations is limited. By leveraging real-world data (RWD), this study helps bridge this gap, supplementing RCT evidence to inform critical clinical decision-making. The real-world effectiveness data provided here offer valuable insights for optimizing treatment strategies and designing future clinical trials.

## Data Availability

Publicly available datasets were analyzed in this study. This data can be found here: https://www.fda.gov/drugs/development-approval-process-drugs/drug-approvals-and-databases.
