# Supplementary material for: Adverse events associated with ustekinumab in Crohn's disease treatment: an analysis based on the FAERS database
Source: Front Med (Lausanne). 2025 Nov 6;12:1657247. doi: 10.3389/fmed.2025.1657247 (PMC12631257; doi:10.3389/fmed.2025.1657247)
Supplement: Supplementary file 1 [file Table_1.docx]

# SUPPORTING INFORMATION

# Additional Tables

**Table S1 Four table of measure of disproportionality**

**Table S2 ROR, PRR, BCPNN and MGPS methods, formulas, and thresholds**

**Table S3 The top 30 PTs of ustekinumab in Crohn's disease ranked by number of reports using disproportionality analysis in FAERS database**

**Table S4 The top 30 PTs of ustekinumab in Crohn's disease ranked by ROR using disproportionality analysis in FAERS database**

## Table S1 Four table of measure of disproportionality

|  | **Target AEs reported** | **Non-target AEs reported** | **Total** |
| --- | --- | --- | --- |
| Drug | a | b | a + b |
| Non-drugs | c | d | c + d |
| Total | a + c | b + d | N = a + b + c + d |

a = the number of reports of ustekinumab with the adverse event of interest.

b = the number of reports of all other drugs with the adverse event of interest.

c = the number of reports of ustekinumab with all other adverse events.

d = the number of reports of all other drugs with all other adverse events.

Abbreviations: AE, adverse event.

## Table S2 ROR, PRR, BCPNN and EBGM methods, formulas, and thresholds

| **Method** | **Calculation formula** | **Threshold** |
| --- | --- | --- |
| ROR | 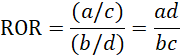    95%CI=*e*^1n(ROR)±1.96SE^ | a ≥ 3  ROR ≥ 3  95% CI (lower limit) > 1 |
| PRR | $PRR=\frac{\text{a}/\text{（}a+b\text{）}}{c/(c+d)}$ $SE\text{（}1\text{n}PRR\text{）}=\sqrt{(\frac{1}{\text{a}}-\frac{1}{a+b}+\frac{1}{c}-\frac{1}{c+d})}$    95%CI=*e*^1n(PRR)±1.96SE^ | a ≥ 3  PRR ≥ 2  95% CI (lower limit) > 1 |
| BCPNN | $IC=$ 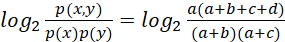  E(IC)=${log}_{2}\frac{(a+\gamma11)(a+b+c+d+\alpha)(a+b+c+d+\beta)}{（a+b+c+d+\gamma）(a+b+\alpha1)(a+c+\beta1)}$  V(IC) = 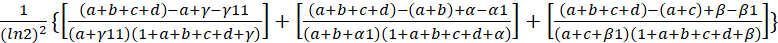  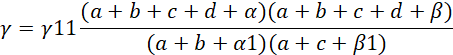    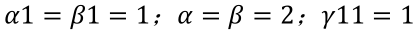 | IC025 > 0 |
| EBGM | 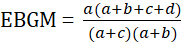  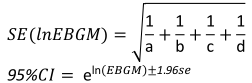 | EBGM05 > 2 |

a = the number of reports of ustekinumab with the adverse event of interest.

b = the number of reports of all other drugs with the adverse event of interest.

c = the number of reports of ustekinumab with all other adverse events.

d = the number of reports of all other drugs with all other adverse events.

Abbreviations: ROR, reporting odds ratio; PRR, proportional reporting ratio; BCPNN, bayesian confidence propagation neural network; EBGM, empirical Bayesian geometric mean; CI, confidence interval; IC, information component; IC025, the lower limit of the 95% one-sided CI, of the IC; EBGM05, the lower 95% one-sided CI, of EBGM.

## Table S3 The top 30 PTs of ustekinumab in Crohn's disease ranked by number of reports using disproportionality analysis in FAERS database

| PTs | AE  Reports | ROR  (95% Cl） | PRR  (95% Cl） | BCPNN  IC (IC025) | EBGM  (EBGM05) |
| --- | --- | --- | --- | --- | --- |
| Off label use | 3,110 | 4.11(3.96, 4.26) | 3.89(3.74, 4.05) | 1.95(1.9) | 3.87(3.76) |
| Crohn's disease | 2,743 | 64.06(61.53, 66.69) | 60.15(57.84, 62.55) | 5.79(5.73) | 55.21(53.38) |
| Product dose omission issue | 1,721 | 7.26(6.92, 7.62) | 7.02(6.75, 7.3) | 2.8(2.73) | 6.95(6.68) |
| Product use issue | 1,394 | 8.68(8.23, 9.16) | 8.44(7.96, 8.95) | 3.06(2.98) | 8.35(7.98) |
| Inappropriate schedule of product administration | 917 | 5.26(4.92, 5.61) | 5.17(4.87, 5.48) | 2.36(2.27) | 5.14(4.86) |
| Abdominal pain | 694 | 4.42(4.1, 4.77) | 4.37(4.04, 4.73) | 2.12(2.01) | 4.35(4.08) |
| Lower respiratory tract infection | 479 | 13.54(12.37, 14.83) | 13.41(12.16, 14.79) | 3.72(3.59) | 13.16(12.2) |
| Infusion related reaction | 453 | 8.99(8.19, 9.87) | 8.91(8.08, 9.83) | 3.14(3) | 8.8(8.14) |
| Accidental exposure to product | 353 | 4.11(3.7, 4.57) | 4.09(3.71, 4.51) | 2.02(1.87) | 4.07(3.73) |
| Product storage error | 327 | 3.99(3.58, 4.45) | 3.97(3.53, 4.47) | 1.98(1.82) | 3.95(3.61) |
| Therapeutic response decreased | 289 | 7.26(6.46, 8.15) | 7.22(6.42, 8.12) | 2.84(2.67) | 7.15(6.49) |
| Abscess | 282 | 25.36(22.51, 28.58) | 25.21(22.41, 28.36) | 4.6(4.43) | 24.32(22.01) |
| Intestinal obstruction | 269 | 10.01(8.87, 11.29) | 9.95(8.85, 11.19) | 3.3(3.12) | 9.82(8.88) |
| Exposure during pregnancy | 266 | 5.01(4.44, 5.66) | 4.99(4.44, 5.61) | 2.31(2.14) | 4.96(4.48) |
| Clostridium difficile infection | 264 | 14.37(12.72, 16.24) | 14.29(12.7, 16.07) | 3.81(3.63) | 14.01(12.65) |
| Underdose | 227 | 3.53(3.09, 4.02) | 3.51(3.06, 4.03) | 1.81(1.62) | 3.5(3.14) |
| Therapeutic product effect decreased | 207 | 4.12(3.59, 4.72) | 4.1(3.57, 4.7) | 2.03(1.83) | 4.09(3.64) |
| Fistula | 199 | 25.69(22.29, 29.6) | 25.58(22.3, 29.34) | 4.62(4.42) | 24.66(21.9) |
| Haematochezia | 193 | 4.41(3.82, 5.08) | 4.39(3.83, 5.04) | 2.13(1.92) | 4.37(3.88) |
| Anal abscess | 174 | 40.66(34.88, 47.4) | 40.5(34.62, 47.38) | 5.26(5.04) | 38.22(33.62) |
| Frequent bowel Movements | 160 | 7.6(6.5, 8.88) | 7.57(6.47, 8.86) | 2.91(2.68) | 7.5(6.58) |
| Drug level decreased | 147 | 16.71(14.18, 19.68) | 16.65(14.23, 19.48) | 4.02(3.79) | 16.27(14.18) |
| Cellulitis | 132 | 3.59(3.03, 4.27) | 3.59(3.01, 4.28) | 1.84(1.59) | 3.57(3.1) |
| Kidney infection | 124 | 7.97(6.68, 9.52) | 7.95(6.66, 9.48) | 2.98(2.72) | 7.87(6.78) |
| Nephrolithiasis | 114 | 3.36(2.8, 4.04) | 3.36(2.82, 4.01) | 1.74(1.48) | 3.35(2.87) |
| Intestinal stenosis | 102 | 35.43(29.03, 43.25) | 35.35(29.06, 43) | 5.07(4.78) | 33.61(28.44) |
| Abdominal abscess | 84 | 28.58(22.97, 35.57) | 28.53(23, 35.39) | 4.78(4.46) | 27.39(22.81) |
| Gastrointestinal infection | 79 | 11.95(9.57, 14.93) | 11.93(9.62, 14.8) | 3.55(3.23) | 11.74(9.74) |
| Skin cancer | 78 | 3.77(3.02, 4.71) | 3.77(3.04, 4.68) | 1.91(1.59) | 3.75(3.11) |
| Postoperative wound infection | 76 | 13.73(10.94, 17.23) | 13.71(10.84, 17.35) | 3.75(3.42) | 13.45(11.12) |

Abbreviations: PT, preferred term; AE, adverse event; FAERS, the U.S. Food and Drug Administration Adverse Event Reporting System; ROR, reporting odds ratio; CI, confidence interval; PRR, proportional reporting ratio; BCPNN, Bayesian confidence propagation neural network; IC, information component; EBGM, empirical Bayesian geometric mean.

## Table S4 The top 30 PTs of ustekinumab in Crohn's disease ranked by ROR using disproportionality analysis in FAERS database

| PTs | AE  Reports | ROR  (95% Cl） | PRR  (95% Cl） | BCPNN  IC (IC025) | EBGM  (EBGM05) |
| --- | --- | --- | --- | --- | --- |
| Congenital pulmonary airway malformation | 3 | 116.59(34.17, 397.85) | 116.58(33.91, 400.77) | 6.63(5.09) | 99.24(35.54) |
| Transitional cell carcinoma recurrent | 3 | 99.1(29.45, 333.51) | 99.09(29.4, 334.03) | 6.43(4.9) | 86.3(31.26) |
| Spirochaetal infection | 3 | 90.09(26.96, 301.01) | 90.08(26.72, 303.66) | 6.31(4.79) | 79.39(28.93) |
| Faecal calprotectin | 4 | 82.58(29.2, 233.53) | 82.58(29.22, 233.36) | 6.2(4.85) | 73.51(30.81) |
| Infected fistula | 36 | 80.14(56.7, 113.27) | 80.08(56.27, 113.96) | 6.16(5.67) | 71.53(53.55) |
| External ear cellulitis | 6 | 66.07(28.54, 152.93) | 66.06(28.44, 153.45) | 5.91(4.79) | 60.15(29.8) |
| Crohn's disease | 2,743 | 64.06(61.53, 66.69) | 60.15(57.84, 62.55) | 5.79(5.73) | 55.21(53.38) |
| Periumbilical abscess | 3 | 61.94(18.97, 202.27) | 61.93(19.11, 200.74) | 5.83(4.34) | 56.71(21.07) |
| Anal fistula infection | 8 | 56.84(27.6, 117.03) | 56.83(27.52, 117.36) | 5.71(4.73) | 52.41(28.64) |
| Small intestine adenocarcinoma | 7 | 55.06(25.47, 119.05) | 55.05(25.63, 118.23) | 5.67(4.63) | 50.89(26.7) |
| Rectal abscess | 67 | 54.32(42.34, 69.7) | 54.24(42.04, 69.98) | 5.65(5.29) | 50.2(40.75) |
| Abscess intestinal | 70 | 49.38(38.72, 62.96) | 49.3(38.97, 62.37) | 5.52(5.17) | 45.95(37.49) |
| Incision site abscess | 7 | 48.68(22.59, 104.9) | 48.68(22.67, 104.55) | 5.5(4.47) | 45.41(23.89) |
| Bartholin's abscess | 3 | 47.19(14.63, 152.25) | 47.19(14.56, 152.96) | 5.46(3.99) | 44.11(16.55) |
| Jejunal stenosis | 3 | 46.09(14.3, 148.58) | 46.09(14.22, 149.4) | 5.43(3.96) | 43.15(16.2) |
| Postoperative abscess | 21 | 44.34(28.5, 68.98) | 44.32(28.24, 69.56) | 5.38(4.76) | 41.6(28.74) |
| Anastomotic stenosis | 10 | 44.05(23.22, 83.56) | 44.04(23.06, 84.09) | 5.37(4.49) | 41.35(24.2) |
| Anal abscess | 174 | 40.66(34.88, 47.4) | 40.5(34.62, 47.38) | 5.26(5.04) | 38.22(33.62) |
| Vaginal fistula | 10 | 37.54(19.85, 71) | 37.54(19.66, 71.68) | 5.15(4.28) | 35.57(20.87) |
| Omphalitis | 9 | 36.71(18.76, 71.83) | 36.7(18.85, 71.46) | 5.12(4.2) | 34.82(19.86) |
| Perineal abscess | 14 | 36(21.02, 61.65) | 35.99(21.2, 61.1) | 5.1(4.34) | 34.18(21.79) |
| Intestinal stenosis | 102 | 35.43(29.03, 43.25) | 35.35(29.06, 43) | 5.07(4.78) | 33.61(28.44) |
| Choroid melanoma | 3 | 35.39(11.08, 113.08) | 35.39(11.13, 112.49) | 5.07(3.61) | 33.64(12.73) |
| Pyoderma | 14 | 35.31(20.62, 60.46) | 35.3(20.79, 59.92) | 5.07(4.32) | 33.56(21.4) |
| Abscess rupture | 8 | 34.32(16.86, 69.87) | 34.32(16.95, 69.5) | 5.03(4.06) | 32.67(18.03) |
| Abdominal wall abscess | 13 | 32.66(18.71, 57.01) | 32.65(18.86, 56.52) | 4.96(4.19) | 31.16(19.55) |
| Vaginal abscess | 6 | 31.21(13.76, 70.79) | 31.21(13.7, 71.09) | 4.9(3.8) | 29.85(15.04) |
| Arthritis enteropathic | 7 | 30.03(14.08, 64.06) | 30.03(13.98, 64.5) | 4.85(3.82) | 28.77(15.26) |
| Abdominal abscess | 84 | 28.58(22.97, 35.57) | 28.53(23, 35.39) | 4.78(4.46) | 27.39(22.81) |
| Stoma site abscess | 6 | 26.43(11.69, 59.77) | 26.42(11.6, 60.18) | 4.67(3.58) | 25.45(12.86) |

Abbreviations: PT, preferred term; AE, adverse event; FAERS, the U.S. Food and Drug Administration Adverse Event Reporting System; ROR, reporting odds ratio; CI, confidence interval; PRR, proportional reporting ratio; BCPNN, Bayesian confidence propagation neural network; IC, information component; EBGM, empirical Bayesian geometric mean.
